# Characterisation of carbon nanotubes in the context of toxicity studies

**DOI:** 10.1186/1476-069X-8-S1-S3

**Published:** 2009-12-21

**Authors:** Deborah Berhanu, Agnieszka Dybowska, Superb K Misra, Chris J Stanley, Pakatip Ruenraroengsak, Aldo R Boccaccini, Teresa D Tetley, Samuel N Luoma, Jane A Plant, Eugenia Valsami-Jones

**Affiliations:** 1 Department of Mineralogy, Natural History Museum, Cromwell Road, London, SW7 5BD, UK; 2 Department of Materials, Imperial College London, South Kensington Campus, London, SW7 2AZ, UK; 3 National Heart and Lung Institute, Imperial College London, Dovehouse Street, London, SW3 6LY, UK; 4 U.S. Geological Survey, 345 Middlefield Rd, MS496, Menlo Park, CA 94025, USA; 5 Department of Zoology, Natural History Museum, Cromwell Road, London, SW7 5BD, UK; 6 Department of Earth Science and Engineering, Imperial College London, Exhibition Road, London, SW7 2AZ, UK

## Abstract

Nanotechnology has the potential to revolutionise our futures, but has also prompted concerns about the possibility that nanomaterials may harm humans or the biosphere. The unique properties of nanoparticles, that give them novel size dependent functionalities, may also have the potential to cause harm. Discrepancies in existing human health and environmental studies have shown the importance of good quality, well-characterized reference nanomaterials for toxicological studies.

Here we make a case for the importance of the detailed characterization of nanoparticles, using several methods, particularly to allow the recognition of impurities and the presence of chemically identical but structurally distinct phases. Methods to characterise fully, commercially available multi-wall carbon nanotubes at different scales, are presented.

## Introduction

Nanoparticles are entities that have one, two or three dimensions of 100 nm or less. In recent years, their applications have expanded exponentially in diverse fields, ranging from medicine to electronics, due to their enhanced or novel physicochemical properties. As particles become smaller, the ratio of surface atoms to inner atoms increases giving a higher surface area to nanoparticles compared to their bulk counterparts. The physicochemical properties of the particles are also different from the bulk form and can even be size dependant. Therefore, nanomaterials can be made with special characteristics for dedicated applications. Whilst they are already present in many commercially available products, doubts have been expressed over their safety, given that the same factors responsible for their novel properties may be the source of their potential hazard. At present, commercially available nanomaterials are characterised only for the purpose of establishing whether a desired property is exhibited; there is no legal requirement for toxicity tests.

Carbon nanotubes (CNTs), cylindrical carbon molecules with uniquely high length to diameter ratios, can exist as single-walled (SWCNT) or multi-walled (MWCNT) entities. Their main technological advantage is their exceptional strength, but they also display other interesting physical properties. For these reasons, they have been studied extensively and are already used in many new commercial products. However, their high length to diameter ratios mean they are fibre shaped, and thus have been compared to asbestos. Some studies have found evidence that at least some types of CNTs can, under certain circumstances, show pathogenicity [1]. A further toxicity concern for CNTs and other carbon-based nanomaterials (e.g. fullerenes) is the potential presence of metal impurities, which are the by-product of the synthesis methods [2], as well as the use of surfactants to disperse them in aqueous media, which can modify their properties and hence toxicity as well as interfere directly with the toxicity tests [3].

Here, we make a case for detailed characterisation of CNTs prior to toxicity tests, particularly because manufacturers' characterisation are often incomplete. We demonstrate the presence of carbon based micron-sized structures, effectively structural impurities within MWCNTs, from two different manufacturers; both materials have been used in many previous studies. We emphasis the need to combine characterisation methods and look for impurities at different scales.

## Materials and methods

Multi-walled carbon nanotubes were purchased from Nanocyl and Cheap Tubes Inc. and used as received.

A variety of methods were used for characterisation. Electron microscopy was used for size and shape determination. Transmission electron microscopy (TEM) data were collected from a HITACHI H7100, operating at 100 kV and Scanning Electron Microscopy (SEM) from a Philips XL-30 operating at 5 kV. Optical microscopy was performed using a Zeiss Axioplan micrsocope, set-up for reflected light with JVC digital camera (model KYF70) linked to Automontage imaging software. X-Ray Diffraction (XRD) was used to determine the crystal structures. XRD data were collected using a Nonius PDS 120 powder diffraction system, position sensitive detector (PSD). Measurements were made in reflection geometry with the sample surface at an angle of 3.5° to the incident beam (conditions: Cu K_α1_, 45 kV and 32 mA).

Suspensions were made in a mixture of acetone and water for microscopy characterisation or in acetone for XRD characterisation. A drop was then allowed to dry on suitable substrates: aluminium stub for SEM, carbon coated copper grid for TEM, glass slide for optical microscopy and quartz substrate for XRD.

## Results and discussion

**Figure 1:**
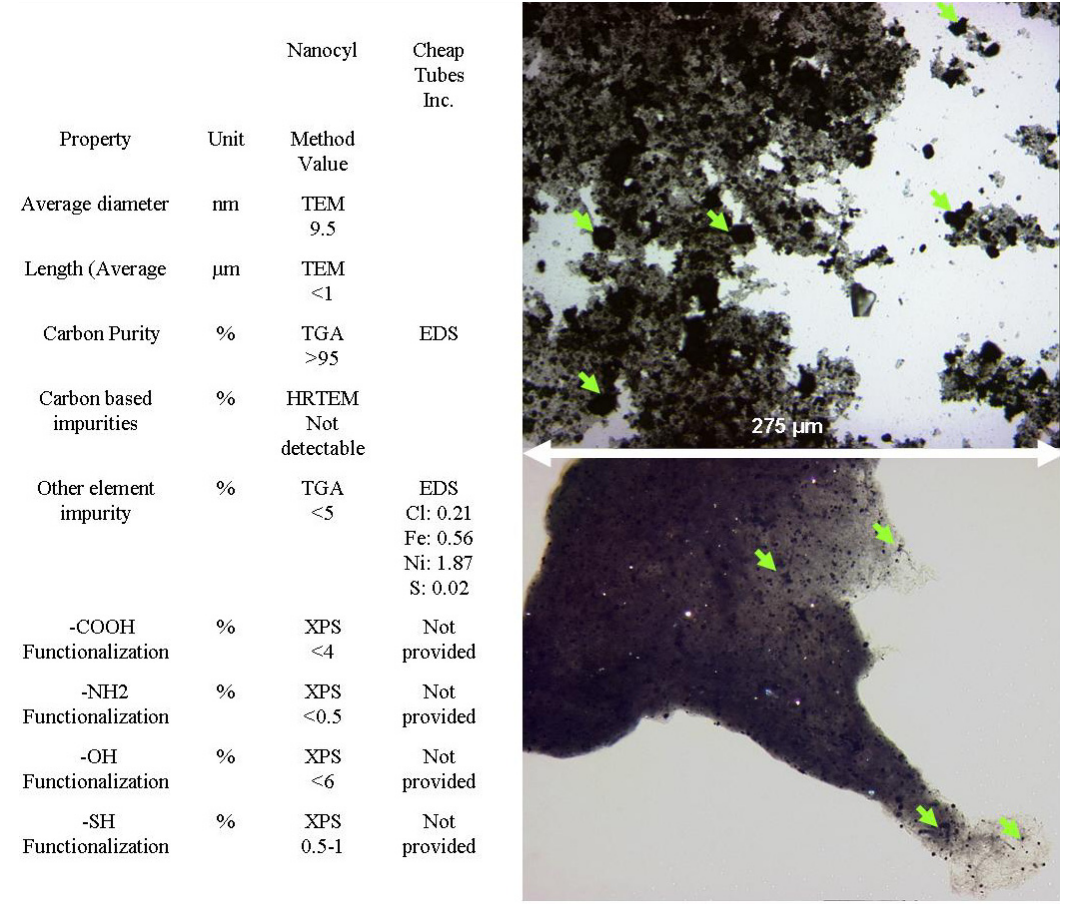
**Characterisation of MWCNTs**. a) Table summarising characterisation data provided by manufacturers. b) Optical microscopy images of CT-MWCNTs (top) and NC-MWCNTs (bottom) showing the presence of microstructures. The width of each image represents 275 μm.

**Figure 2:**
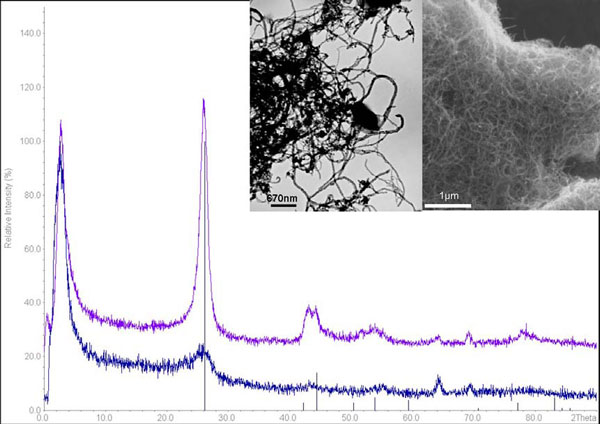
**XRD pattern and corresponding TEM and SEM images of MWCNTs**. The TEM image was obtained for CT-MWCNTs and corresponds to the purple pattern, while the SEM image was obtained for NC-MWCNTs and corresponds to the blue pattern. The peak positions of graphite-2H (ICDD 1071-4630) are indicated in black on the XRD pattern.

Two MWCNT samples, one from Nanocyl (NC-MWCNTs) and one from Cheap Tubes Inc. (CT-MWCNTs) were characterised in this study. According to the manufacturers, nanotubes are tangled together and have a distribution of diameters between 10-40 nm and 30-50 nm respectively. Further details are given on the presence of carbon purity (>95% and 97.34 respectively). Elemental impurities (metal or metal oxides in this case) are present, but at low concentrations (see Figure 1a) and are not considered in this paper. This characterisation is based on techniques such as microanalysis, TGA or ICP, which can provide very accurate chemical information. Unfortunately, these techniques do not provide any information on the crystal structure, which is a particular problem for materials that can exist in structurally different but chemically identical forms. In other words, the samples here contain above 95% of carbon based material but different types of these materials could coexist: fullerene, nanotube, graphite, diamond or amorphous carbon; these cannot be chemically distinguished, and other techniques have to be used to address this issue. XRD is an appropriate tool for the recognition of different types of structures. The presence of graphite impurities was first suspected during XRD characterisation, when the peaks corresponding to graphite were sharper than expected, indicating larger sizes of crystalline structures (Figure 2). XRD detection limit is difficult to define precisely. Depending on the degree of crystallinity, it is estimated to range between 5% for poorly crystalline phases and 1% for well-crystallised phases [4]. The presence of other structurally amorphous carbon based materials is also possible. A peak broadening observed on the XRD pattern for NC-MWCNTs (Figure 2) indicates smaller sizes structures compared to the CT-MWCNTs, although this may have been to some extent due to lattice strain.

Electron microscopy imaging gives valuable details on materials structures but the recognition of nano-impurities made of the same compound can be highly challenging. As indicated by the manufacturers (Figure 1), carbon based impurities are not detectable by high resolution TEM. However, TEM is based on the transmission of electrons through a sample. As a result, the darker the area is, the thicker the sample is on that point. In the case of tangled MWCNTs, it is very hard to differentiate between dark areas where the thickness is due to the fact that the nanotubes are tangled or to the presence of other micro-sized materials. While in SEM, only the surface of the sample is analysed and the structures caged within the MWCNTs will not be imaged.

Based on our experience, electron microscopy provides detailed information at the smallest scales but larger scale investigation can provide complementary information and is as important. In this case, the use of optical microscopy was essential to identify graphite visually (Figure 1) and therefore give support to the XRD data. The ability to acquire a large-scale view of the sample by "zooming-out" can be very helpful even when the material for characterisation is nanostructured. Optical microscopy imaging can be described as equivalent to TEM imaging, but at lower resolution. There, the MWCNTs are almost transparent to the light beam and the graphitic microstructures in the sample become obvious.

## Conclusion

Detailed characterisation of engineered nanomaterials is essential to establish whether there is a link between the novel physicochemical properties exhibited by the materials and toxicity. Impurities, as shown here for MWCNTs, may exist at different scales, and can be "invisible" to some analytical methods. Characterisation at a range of scales, using different instrumentation and probing different material properties is essential before embarking on toxicological studies.

## Note

The peer review of this article can be found in Additional file 1.

## Competing interests

The authors declare that they have no competing interests.

## Authors' contributions

All authors contributed to this work by discussing the results and implications and commenting on the manuscript at all stages. A.R.B, J.A.P, E.V.J, S.N.L and T.D.T conceived the project; E.V.J is the PI. A.R.B, E.V.J, and T.D.T supervised the project. D.B designed and performed experiments, analysed data and wrote the paper; A.D, S.K.M and P.R. helped designing experiments and contributed to paper writing. C.J.S. performed optical microscopy analysis. All authors discussed the results and their implications and commented on the manuscript at all stages.  

## Supplementary Material

Additional file 1 Peer review. Click here for file
