# Supplementary material for: Characterisation of carbon nanotubes in the context of toxicity studies
Source: Environ Health. 2009 Dec 21;8(Suppl 1):S3. doi: 10.1186/1476-069X-8-S1-S3 (PMC2796499; doi:10.1186/1476-069X-8-S1-S3)
Supplement: Additional file 1 — Peer review. [file 1476-069X-8-S1-S3-S1.doc]

| Article ref no. | A1471-2105X SZ S3 |
| --- | --- |
| Title | Characterisation of carbon nanotubes in the context of toxicity studies |
| Author(s) | Berhanu et al. |

**Authors**

We thank both referees for their comments. Referee #2 gave us the opportunity to clarify some points in the manuscript as follows:

**General comments:**

Comment 1: Here the focus is on crystallinity. I accept that the authors have identified graphitic structures within carbon nanotubes but the evidence they present for the unambiguous identification of graphite patches is less than wholly convincing, although the XRD data do indicate its presence. What is the implication of the graphitic phase for toxicity? Or is it just important to point out that there are structural varieties that may have unknown toxic consequences?

Answer: XRD is a method that cannot provide, "false positives", i.e. if graphite is identified, it is unambiguously present. In terms of toxicity, the main point of this communication is to show how characterisation of nanomaterials has to be adapted to nanotoxicity. The presence of graphite can increase toxicity or inflammatory responses, as any insoluble or sparingly soluble material could.

Action: None needed.

Comment 2: The manufacturer-provided data indicate quite high levels of transition metals but the implications are not discussed; rather their focus is on structural “impurities”. Could the metals be bioavailable? It might be better for clarity in this short paper to restrict description and discussion to the crystalline material and leave these metal contaminants to another discussion.

Answer: Here, we chose to deal with crystal structure issues only. The presence of metals in MWCNT samples is well-known as they are used as catalysts during production and indeed the subject of another manuscript we are currently preparing. We agree that the discussion should here focus on crystalline structures, and have clarified this in our revised version.

Action: Modified on the revised version.

**Major compulsory revisions:**

Page 1: “Addresses”

Introduction, paragraph 3: “Here, we make a case for the need to a detailed characterisation of CNTs prior to toxicity tests, particularly because manufacturers’ characterisation can be incomplete.” Is rather cumbersome. I suggest “Here, we make a case for detailed characterisation of CNTs prior to toxicity tests, particularly because manufacturers’ characterisation are often incomplete.”

Action: Sentence has been modified on the revised version.

Results and discussion

Paragraph 1, line 11: change type to types.

Paragraph 1, lines 18-19: Any reference for these detection limits?

Action: Reference has been added on the revised version.

Paragraph 1, lines 19-21: “A peak broadening observed on the XRD pattern … indicates smaller sizes structures ...” Smaller crystallite size is likely but might peak broadening also be related to lattice strain?

Answer: The XRD data obtained for larger diameter MWCNTs from the same manufacturers shows sharper peaks. Also, the pattern obtained for the sample after grinding of the MWCNTs does not show any broadening difference and it would have if their was lattice strain in the sample. Hence, we concluded that the peak broadening was mainly due to crystal size but it can be pointed out that lattice strain can also affect it.

Action: Sentence has been modified in the reviewed version.

Paragraph 1, line 27: what do you mean by bigger?

Answer: The opacity of the granules indicates thicker areas that are not transparent to the light beam. Hence, the thickness of the sample on these points is of micron scale.

Paragraph 2, lines 7-8: How are you sure these optical features are graphite? Is spectral reflectance possible?

Answer: These features could only be identified as graphite by default, when compared to the XRD data. The grain size of the particles precludes the use of spectral reflectance to determine whether graphite is present. Diffuse reflectance would probably not be diagnostic in itself, giving an averaged and rather general carbon signature.

Fig 1. Not clear from the text why optical images reveal the presence of microstructures and you need to clarify what the term “microstructure” means in this context.

Answer: Microstructure can be defined as any structure that is in the micron-scale in all three dimensions.

Reference 2: all names have an extra character at the end.

Action: Modified on the revised version.
